# Seven and up: individual differences in male voice fundamental frequency emerge before puberty and remain stable throughout adulthood

**DOI:** 10.1098/rsos.160395

**Published:** 2016-10-05

**Authors:** Meddy Fouquet, Katarzyna Pisanski, Nicolas Mathevon, David Reby

**Affiliations:** 1 Mammal Vocal Communication and Cognition Research Group, School of Psychology, University of Sussex, Brighton, UK; 2 Equipe Neuro-Ethologie Sensorielle, ENES/Neuro-PSI CNRS UMR 9197, University of Lyon/Saint-Etienne, Saint-Etienne, France

**Keywords:** voice pitch, fundamental frequency, longitudinal study, non-verbal communication, pubertal, testosterone

## Abstract

Voice pitch (the perceptual correlate of fundamental frequency, *F*0) varies considerably even among individuals of the same sex and age, communicating a host of socially and evolutionarily relevant information. However, due to the almost exclusive utilization of cross-sectional designs in previous studies, it remains unknown whether these individual differences in voice pitch emerge before, during or after sexual maturation, and whether voice pitch remains stable into adulthood. Here, we measured the *F*0 parameters of men who were recorded once every 7 years from age 7 to 56 as they participated in the British television documentary *Up Series*. Linear mixed models revealed significant effects of age on all *F*0 parameters, wherein *F*0 mean, minimum, maximum and the standard deviation of *F*0 showed sharp pubertal decreases between age 7 and 21, yet remained remarkably stable after age 28. Critically, men's pre-pubertal *F*0 at age 7 strongly predicted their *F*0 at every subsequent adult age, explaining up to 64% of the variance in post-pubertal *F*0. This finding suggests that between-individual differences in voice pitch that are known to play an important role in men's reproductive success are in fact largely determined by age 7, and may therefore be linked to prenatal and/or pre-pubertal androgen exposure.

## 1. Introduction

As the human body develops and matures throughout the lifespan, anatomical and physiological modifications are reflected in the voice ([1] for review). Most notably, there is a sizeable shift in mean voice pitch (the perceptual correlate of fundamental frequency, *F*0) following puberty in males [2–4]. This acute pubertal drop in male *F*0, perceived as a salient decrease in voice pitch, is caused by a surge in circulating androgen levels that is several times greater in males than females and that disproportionately increases the length of male vocal folds by 60%, dramatically decreasing their rate of vibration [3,5]. Although *F*0 also decreases during adolescence in females, the drop is proportionate and comparatively small [6], resulting in the highly sexually dimorphic pitch that characterizes adult human voices [7].

A growing body of research suggests that men's *F*0 has undergone intense sexual selection to advertise masculinity, mate quality and threat potential [7]. Dozens of studies indicate that men with relatively low-pitched voices are judged as more attractive by female listeners ([8] for review) and enjoy higher reproductive success (e.g. report higher numbers of sexual partners [9] and have more children born to them in a natural fertility population [10]). Men with low-pitched voices are also judged as physically larger and stronger, more socially dominant, and more competent than are men with higher pitched voices ([11] for review). Although individual differences in men's *F*0 are only weakly tied to differences in physical body size [12], men with relatively low *F*0 have higher circulating levels of testosterone [5,7,13–15] and are indeed more formidable than are men with higher *F*0 [16].

Research to date is therefore consistent with the hypothesis that men's *F*0 is an androgen-mediated secondary sexual characteristic. Studies examining the development of analogous, androgen-mediated cues in men's faces suggest that masculinized facial features remain fairly stable throughout the lifetime and emerge before the onset of puberty [17–19]. For instance, masculinized facial features in adult men correlated with their prenatal testosterone levels measured from umbilical cord blood [19]. Men with masculinized faces typically have lower pitched voices, suggesting that similar (e.g. hormonal) mechanisms may operate on the development of both facial masculinity and male *F*0 [20].

Yet, the extent to which individual differences in men's voice *F*0 remain stable throughout adulthood, or potentially emerge even before pubertal masculinization of the vocal anatomy, has never been investigated. This is largely due to the inherent difficulty of conducting longitudinal voice research with humans. Hence, researchers examining *F*0 across the lifespan have almost exclusively used cross-sectional cohort designs, comparing the *F*0s of different individuals at different ages (e.g. [2,5,6,21,22]). The few studies using a within-individual design focused on a period of only 1–5 years in children or adolescents [4,23–26], with the exception of one study that compared the same 15 adult women recorded once in their 20s and once in their 60s using archival recordings [27]. Within-individual designs provide the unique opportunity to test for longitudinal stability of vocal traits while controlling for confounding cohort effects and other individual differences, thereby offering a comparatively robust test of the effects of age and life stage on the voice.

Cross-sectional studies suggest that, in addition to the salient difference in *F*0 between adult men and women, there exists considerable variation in *F*0 within sex-age classes. Individual differences in adult *F*0 typically range between 80 and 175 Hz among men, and 160 and 270 Hz among women [1]. Similar variation is present among pre-, peri- and post-pubertal adolescents [3–6,28,29], and even the average *F*0 of three-month-old infants' cries ranges from 350 to 550 Hz between individuals, independently of the babies' sex [30]. These studies thus further hint at the possibility that individual differences in *F*0 might emerge very early in ontogeny.

Using an innovative and relatively untapped resource in voice research (archival recordings), the goals of the present study were twofold. First, we aimed to confirm pubertal and age-related influences on men's *F*0 using a within-subject longitudinal design that spanned 50 years. Second, we aimed to test the hypothesis that individual differences in *F*0, already present before the onset of puberty, predict individual differences in *F*0 after puberty and throughout adulthood.

## 2. Material and methods

### 2.1. Voice sample and *Up Series*

We analysed the voices of all 10 men who took part in the British television documentary the *Up Series* [31]. All were born in England in the year 1956 and were audio–video recorded for the series once every 7 years from age 7 to 56. The *Up Series* was first broadcast in 1964 (*Seven Up!*), with subsequent documentaries debuting in 1970 (*7 Plus Seven*), 1977 (*21 Up*), 1984 (*28 Up*), 1991 (*35 Up*), 1998 (*42 Up*), 2005 (*49 Up*) and 2012 (*56 Up*) [31]. In addition to 10 men, the *Up Series* also followed the lives of four British women, however, due to this small and unrepresentative sample, data from these women were not included in this study.

The original aim of the documentary, in 1964, was to get a glimpse of England at the turn of the millennium, at a time when ‘the shop-stewards and executives of the year 2000 are now seven years old’ [32]. The directors chose children from various socio-economic backgrounds. Each episode of the *Up Series* features interviews in which the participants of the documentary are asked to discuss their personal and professional lives, their values and aspirations, and to share their opinions on a range of topics. These interviews offer a large and longitudinal repertoire of standardized voice recordings.

### 2.2. Audio extraction

Short audio clips of speech were extracted from each *Up Series* DVD and saved as WAV files with Audacity v. 1.2.6. We extracted a total of 300 voice clips with an average duration of 11.3 ± 6.6 s, while documenting the speaker, context and recording conditions (e.g. setting, noise level) of each clip. To standardize these variables and reduce potential contextual or social influences on vocal production, we analysed only voice clips extracted from interviews, as well as those in which background noise was negligible and strong emotional content was absent (257 clips). Hence, clips containing highly emotional (e.g. angry) speech or persistent chaotic vocalizations (e.g. laughing, crying, whispering, shouting) were excluded from acoustic analysis. Multiple voice clips were extracted for each individual at each given age (see table 1 for duration and number of voice clips, and participation record).

**Table 1.** Total duration (and number) of voice clips analysed for each individual at each age. Total duration is given in seconds followed by the total number of clips (in brackets). Grand totals are a sum of these values across all ages for each individual (final column) and across all individuals for each age (bottom row). Blank cells indicate instances in which an individual did not take part in the documentary at that particular age.

	age								
ID	7	14	21	28	35	42	49	56	grand total
1	34.2 (3)	37.7 (5)	53.5 (3)	32.8 (3)	52.9 (4)	28.3 (3)	36.7 (3)	38.0 (4)	314.4 (28)
2	68.9 (5)	43.8 (5)	28.6 (2)	33.9 (3)	51.0 (4)	46.4 (4)	22.0 (2)	57.7 (4)	352.7 (29)
3	34.5 (4)	47.3 (5)	30.0 (2)	77.8 (3)	46.0 (2)	54.4 (2)	24.4 (3)	46.9 (4)	361.6 (25)
4	74.9 (5)	20.5 (4)	57.3 (3)	99.4 (5)	65.7 (5)	37.1 (3)	30.0 (4)	102.6 (6)	487.8 (35)
5	52.8 (4)	39.0 (4)	33.5 (2)	53.8 (5)	32.8 (3)	49.4 (3)	41.8 (3)	60.4 (7)	364.0 (31)
6	14.4 (4)	36.3 (6)	54.5 (4)	88.5 (4)	34.7 (2)	50.5 (4)	55.9 (5)	61.2 (3)	396.4 (32)
7	35.9 (3)	59.3 (5)	16.0 (3)	67.4 (6)		37.6 (3)	21.5 (3)	55.5 (4)	293.6 (27)
8	32.8 (4)	42.4 (6)	70.6 (3)		31.2 (3)		39.4 (3)	55.0 (5)	271.6 (24)
9	18.1 (3)	15.8 (4)	47.5 (4)	37.7 (3)				56.5 (3)	175.8 (17)
10	16.6 (1)	43.8 (5)	38.2 (3)						98.8 (9)
grand total	383.6 (36)	386.3 (49)	430.1 (29)	491.8 (32)	314.5 (23)	303.8 (22)	272.0 (26)	534.2 (40)	3116.7 (257)

### 2.3. Voice analysis

Acoustic editing and analysis were performed in Praat v. 5.2.1 [33]. Following manual removal of fragments of acute noise, multi-voicing and non-verbal vocalizations (e.g. laughter, crying), we used a batch-processing script to measure five parameters of fundamental frequency: mean *F*0 (*F*0_mean_), range (*F*0_min_ and *F*0_max_) and *F*0 contour including standard deviation (*F*0_s.d._) and the coefficient of variation (*F*0_CV_). *F*0_CV_ is given by *F*0_s.d._/*F*0_mean_ and represents the logarithmic perception of voice pitch (i.e. controlling for the effect of magnitude on variability [34]). All *F*0 parameters were measured using Praat's autocorrelation algorithm with a search range of 150–400 Hz (age 7) or 75–300 Hz (ages 14–56) and a time step of 0.01 s. Spurious octave jumps were manually corrected [30]. Perceptually, voices with relatively lower *F*0_mean_*, F*0_min_ and *F*0_max_ will sound lower-pitched or ‘deeper’, whereas voices with relatively lower *F*0_s.d._ and *F*0_CV_ will sound more monotone.

## 3. Results

Linear mixed models (LMMs) fit by maximum-likelihood estimation were used to examine the effect of age on each *F*0 parameter separately. Each model included individual identity as a random subject variable, allowing the intercept to vary between subjects, and age as a fixed factor. The results of our LMMs confirmed that all *F*0 parameters changed significantly with age (table 2).

**Table 2.** Linear mixed models examining the effect of age on fundamental frequency parameters.

	source	d.f._1_, d.f._2_	*F*	*p*-value
*F*0_mean_	intercept	1, 9.2	438.5	<0.001
	age	7, 240.9	286.6	<0.001
*F*0_min_	intercept	1, 8.9	945.8	<0.001
	age	7, 241.9	147.7	<0.001
*F*0_max_	intercept	1, 9.6	836.1	<0.001
	age	7, 242.3	87.4	<0.001
*F*0_s.d._	intercept	1, 9.4	152.3	<0.001
	age	7, 242.1	21.3	<0.001
*F*0_CV_	intercept	1, 9.1	301.2	<0.001
	age	7, 242.5	6.5	<0.001

**Figure 1.**
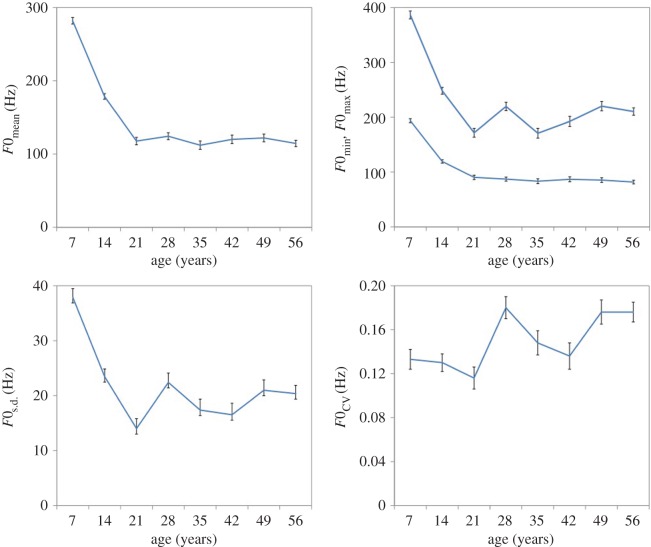
Mean fundamental frequency (*F*0) parameters for males at each age. Error bars represent the standard error of the mean.

Planned pairwise comparisons with Bonferroni's correction confirmed that *F*0_mean_, *F*0_min_, *F*0_max_ and *F*0_s.d._ values dropped significantly and dramatically from age 7 to 14, and again from age 14 to 21 (table 3). These *F*0 parameters then remained relatively stable following puberty, although we observed acute transient increases in *F*0 parameters at age 28. These age-related changes in *F*0 are further illustrated in figure 1.

**Table 3.** Pairwise comparisons of within-individual differences in fundamental frequency parameters at every given age. The cells above the grey median indicate the mean difference in the given *F*0 parameter (in Hertz) measured at two given ages (i.e. subtracting the value at the younger age from the value at the older age), based on estimated marginal means. The corresponding cells below the grey median indicate whether this difference was statistically significant, where *p<0.05 (significant at the 0.05 level) and ***p <0.0009 (significant following Bonferonni's correction for multiple comparisons).

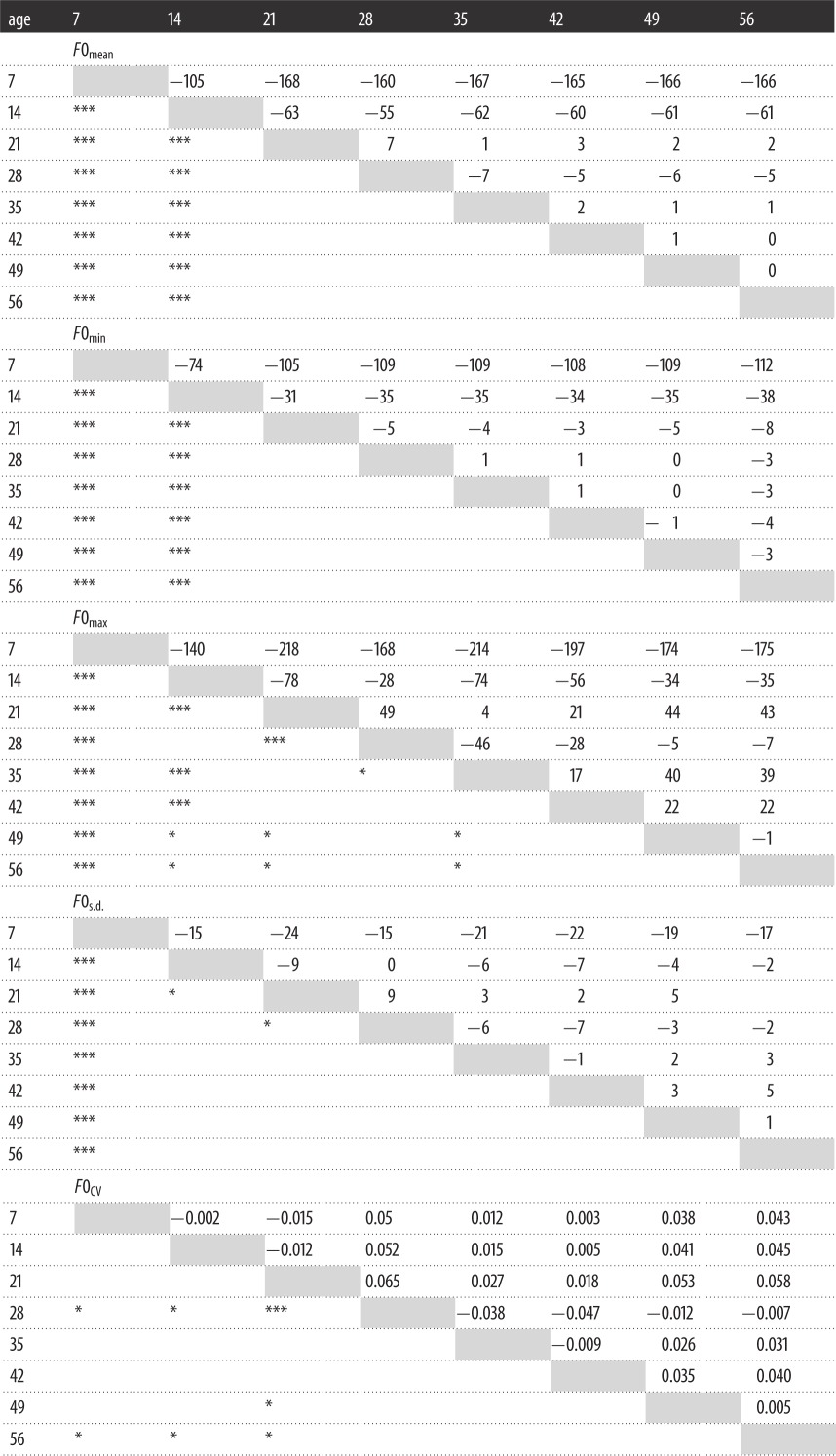

**Figure 2.**
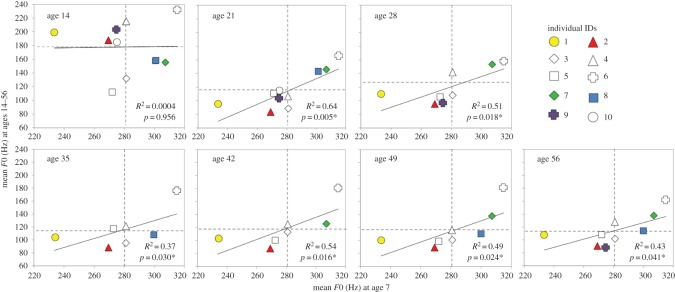
Within-individual relationships between men's *F*0 before puberty (age 7), around the time of puberty (age 14) and after puberty (ages 21–56). Each individual is represented by a unique symbol. Solid lines represent the slope of the linear regression and dashed lines represent the sample average *F*0 measured at each age. *p < 0.05, two-tailed.

Critically, despite significant pubertal effects on *F*0, linear regression models revealed that men's *F*0 at age 7 strongly predicted their *F*0 at every subsequent post-pubertal age in adulthood (ages 21–56, *r*s = 0.61–0.8, *p* < 0.001), however, *F*0 at age 7 did not predict men's *F*0 at the peri-pubertal age of 14 (*r* = 0.02, *p* > 0.05; figure 2). Men's *F*0s at age 7 explained 37–64% of the variance in their *F*0s at ages 21–56 (figure 2).

## 4. Discussion

In this longitudinal study, we tracked men's fundamental frequency (*F*0) over a span of 50 years of their lives. Our results corroborate those of earlier cross-sectional and cohort studies, demonstrating sharp pubertal decreases in male *F*0 parameters between the ages of 7 and 21 [2,3]. Our study contributes two additional key findings. First, despite these profound pubertal drops in *F*0, we demonstrate that men's *F*0 stabilizes after puberty and remains remarkably stable throughout adulthood. Second, our results reveal that individual differences in men's *F*0 are already established by age 7, long before sexual maturation and pubertal influences on the vocal anatomy. These findings have broad implications for our understanding of the developmental mechanisms, adaptive functions and social perception of human voice pitch.

Voice pitch is arguably the most perceptually salient and evolutionarily relevant non-verbal vocal component in humans. Of particular relevance is its close relationship to male dominance and reproductive success, where, for example, men with lower-pitched voices are attributed with higher masculinity or maleness [13] and have greater access to high-quality mates ([1,7,8] for reviews). Our results show that individual differences in men's *F*0 are not temporary, but rather stable throughout adulthood, providing a reliably static signal of men's mate quality. In addition, these stable individual differences emerge in childhood as 64% of the variance in adult men's *F*0 could be explained by their *F*0 before the onset of puberty.

Although hormone levels were not accessible in this study, our findings, combined with those of previous studies demonstrating the effects of testosterone on laryngeal and vocal fold morphology and on subsequent voice pitch production, suggest that pre-pubertal and potentially even prenatal androgen levels may explain a significant portion of the variance in adult *F*0. Pubertal androgens may function to activate the expression of secondary sex traits, such as *F*0, whereas the nature or magnitude of this masculinization may be predetermined very early in ontogeny [35]. Indeed, researchers have found that the second-to-fourth digit ratio (an index of fetal testosterone exposure [36]) and prenatal serum testosterone levels predict the degree of facial masculinization in pre-pubertal boys [17] and adult men [18,19].

We observed an average drop in mean *F*0 of 105 Hz between ages 7 and 14, and an additional 63 Hz drop between ages 14 and 21, far exceeding the just-noticeable differences in voice pitch perception (approx. 5 Hz [37,38]). Minimum, maximum and the standard deviation of *F*0 showed similarly salient decreases at these ages (table 3). These drastic drops in voice pitch parameters are likely to reflect the effects of pubertal androgens on vocal fold lengthening and dynamics [3,5]. Puberty typically begins between age 9 and 14 in British boys and spans approximately 2 years [39], wherein ‘voice breaking’ (an acute drop in *F*0/voice pitch) occurs in the final stages of puberty [4]. While our findings indicate that pre-pubertal *F*0 predicts post-pubertal *F*0, there was no relationship between men's *F*0 before puberty (age 7) and around the time of puberty (age 14). This is probably because, as illustrated in the top-left panel of figure 2, only four individuals had completed pubertal *F*0 lowering by age 14 (those below the 180 Hz average, wherein individual differences in *F*0 ranged between 110 and 230 Hz), whereas by age 21, all individuals' *F*0s had fallen within the characteristic range for adult men (80–160 Hz).

Although our findings demonstrate longitudinal stability in men's average *F*0, this voice feature is known to vary at the intra-individual level as a function of mood and prosodic expression ([40] for review). Hence, all acoustic analyses were performed on speech from interviews that lacked strong emotional content, and *F*0 measures were averaged across multiple interviews for each individual at each age. This allowed us to compute representative, average *F*0 measures for each individual and to largely control for the influence of emotions, such as intense stress or excitement, on voice production.

Intriguingly, we observed a transient but systematic increase in men's *F*0 parameters at age 28. We predict that this brief rise in *F*0 may be related to social life events, such as first marriage or childbirth, which often take place around this age among European men. Indeed, cues to mating and parenting effort may manifest in the voice, either through the behavioural mechanism of volitional voice modulation [41] or through physiological changes in the body (e.g. changes in relative hormone levels [7,13–15]) that could in turn have an indirect effect on vocal production. While there is some evidence of lower testosterone levels in married versus unmarried men and among those with children versus those without (see e.g. [42–44]), it remains unclear whether this reflects between-individual differences or within-individual fluctuation in androgen levels. The potential influence of social life events on vocal production warrants investigation using a larger longitudinal dataset.

The *Up Series* documentary included four women; however, this sample size was too small to be sufficiently representative. Replication studies should, therefore, include women to test whether individual differences in *F*0 also emerge before puberty among females and should include infants as well as post-menopausal/andropausal adults in order to determine whether individual differences in *F*0 are already present at birth and remain stable after the age of 60. While clearly challenging, future studies may also examine the degree to which sex hormone levels, which we were not able to access in this study, correlate with voice *F*0 across the lifespan. In particular, researchers may test whether higher prenatal testosterone levels in males predict lower *F*0 in adulthood.

Our findings have broad social implications. A large body of research demonstrates that voice pitch affects judgements of attractiveness, masculinity, dominance, competence, likeability and trustworthiness, and even impacts political and economic decision-making [45]. Moreover, listeners attribute such traits not only to adults [1] but also to adolescents [5] and even to babies with high- or low-pitched voices [30]. Given that a child's voice pitch hints at the voice pitch he will have as an adult, this attribution of voice-based social labels may begin very early in life, and remain consistent throughout a person's lifetime.

## Data accessibility

The dataset supporting this article has been uploaded to the Sussex Research Online (SRO) repository (Item 63023, http://sro.sussex.ac.uk/id/eprint/63023).
